# Supplementary material for: Reverse vaccinology and subtractive genomics reveal new therapeutic targets against Mycoplasma pneumoniae: a causative agent of pneumonia
Source: R Soc Open Sci. 2019 Jul 31;6(7):190907. doi: 10.1098/rsos.190907 (PMC6689572; doi:10.1098/rsos.190907)
Supplement: Classification tables of potential drug targets for structural quality and evaluation criteria used through the MHOLline program [file rsos190907supp1.docx]

MHOLline Supplementary Table 1: Template ID used by MHOLline for Identified targets for modeling with identity.

| **Protein Name** | **Template ID** | **Idenity (%)** |
| --- | --- | --- |
| WP_010874513.1 | 1pa4 | >=75% |
| WP_010874670.1 | 1n0e | >=75% |
| WP_010874705.1 | 1t71 | >=75% |
| WP_010874779.1 | 2i15 | >=75% |
| WP_014325598.1 | 1zxj | >=75% |

MHOLline Supplementary Table 2: Classification of each sequence in accordance with criteria in table 4.

| **Sequence ID** | **Sequence name** | **MHOLline group name (only G2)** | **Filters Quality** |
| --- | --- | --- | --- |
| 6 | WP_010874513.1 | G2 | Very High |
| 12 | WP_010874670.1 | G2 | Very High |
| 13 | WP_010874705.1 | G2 | Very High |
| 15 | WP_010874779.1 | G2 | Very High |
| 40 | WP_014325598.1 | G2 | Very High |
| 36 | WP_014325566.1 | G2 | High |
| 1 | WP_010874387.1 | G2 | Good |
| 2 | WP_010874410.1 | G2 | Medium to Good |
| 8 | WP_010874577.1 | G2 | Medium to Good |
| 25 | WP_010875025.1 | G2 | Medium to Good |
| 29 | WP_014325292.1 | G2 | Medium to Good |
| 37 | WP_014325567.1 | G2 | Medium to Good |
| 42 | WP_014325643.1 | G2 | Medium to Good |
| 43 | WP_014325646.1 | G2 | Medium to Good |
| 44 | WP_014325666.1 | G2 | Medium to Good |
| 46 | WP_019830475.1 | G2 | Medium to Low |
| 47 | WP_019830488.1 | G2 | Medium to Low |
| 5 | WP_010874483.1 | G2 | Medium to Low |
| 20 | WP_010874917.1 | G2 | Medium to Low |
| 27 | WP_010875045.1 | G2 | Medium to Low |
| 32 | WP_014325427.1 | G2 | Medium to Low |
| 35 | WP_014325526.1 | G2 | Medium to Low |
| 39 | WP_014325596.1 | G2 | Medium to Low |
| 17 | WP_010874873.1 | G2 | Low |
| 48 | WP_019830439.1 | G2 | Low |
| 45 | WP_014325669.1 | G2 | Very Low |

| **Quality** | **Identity** | **Lenght Variation Index** |
| --- | --- | --- |
| Very High | >=75% | <= 0.1 |
| High | >=50% and <75% | <= 0.1 |
| Good | >=50% | > 0.1 and <= 0.3 |
| Medium to Good | >=35% and <50% | <= 0.3 |
| Medium to Low | >=25% and 35% | <= 0.3 |
| Low | >=25% | > 0.3 and <= 0.5 |
| Very Low | >=25% | > 0.5 and <= 0.7 |

MHOLline Supplementary Table 3: MHOLline criteria from G2Group
