# Supplementary figures and images for: Reverse vaccinology and subtractive genomics reveal new therapeutic targets against Mycoplasma pneumoniae: a causative agent of pneumonia
Source: R Soc Open Sci. 2019 Jul 31;6(7):190907. doi: 10.1098/rsos.190907 (PMC6689572; doi:10.1098/rsos.190907)

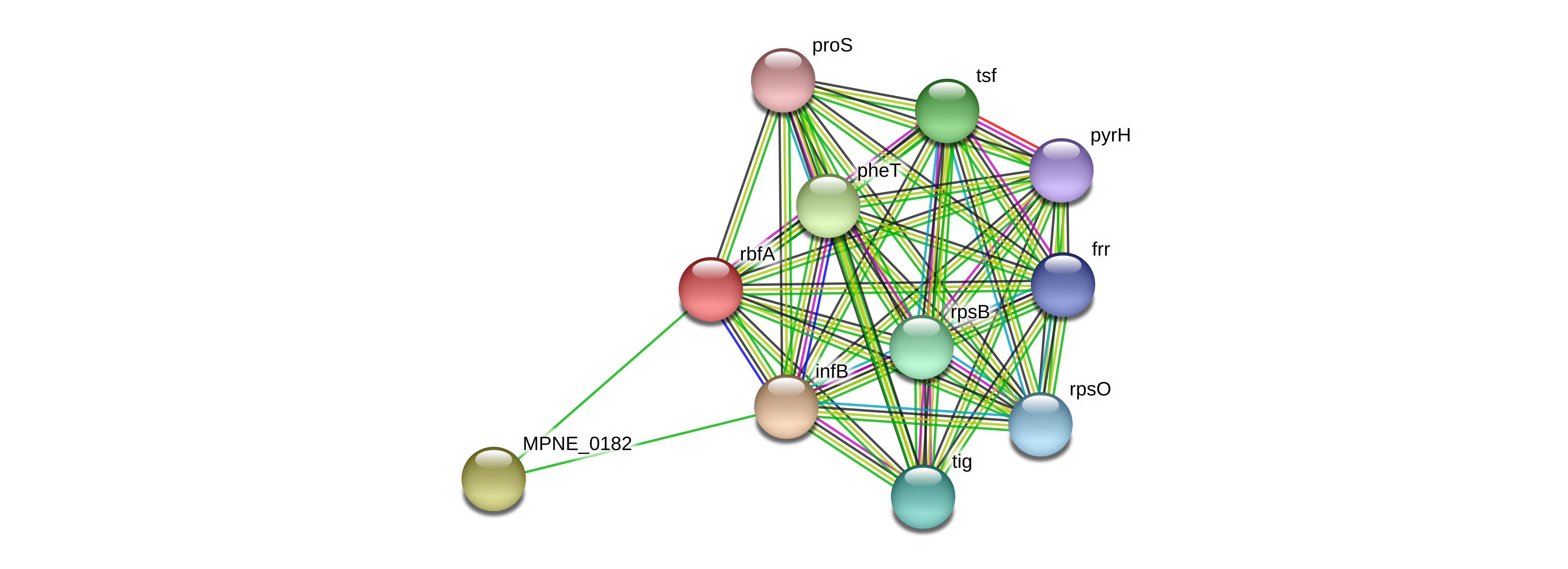

Supplement: Potential drug target Ribosome-binding factor A and its protein interactions from STRING [file rsos190907supp3.png]

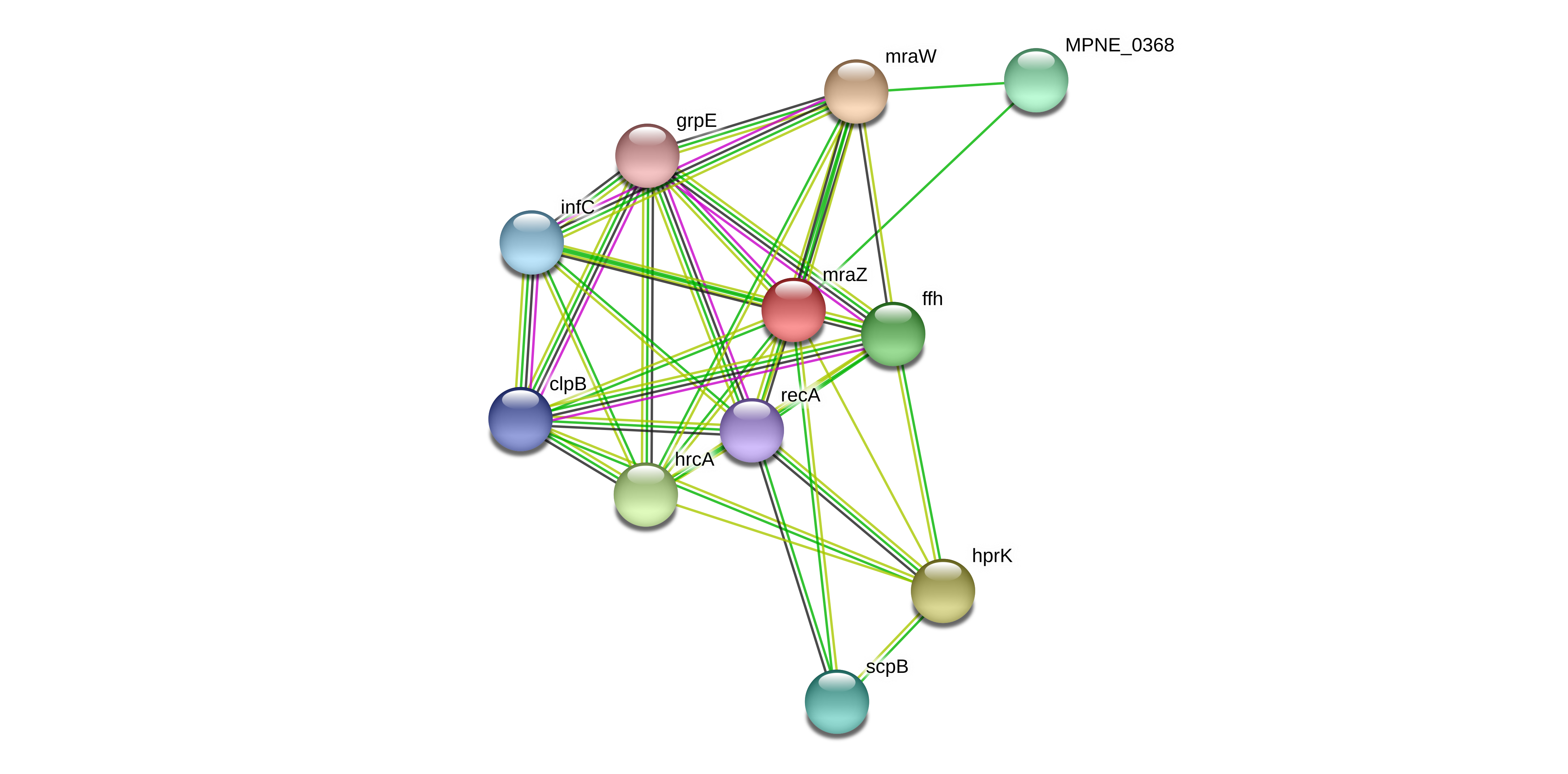

Supplement: Potential drug target Transcriptional regulator MraZ and its protein interactions from STRING [file rsos190907supp5.png]

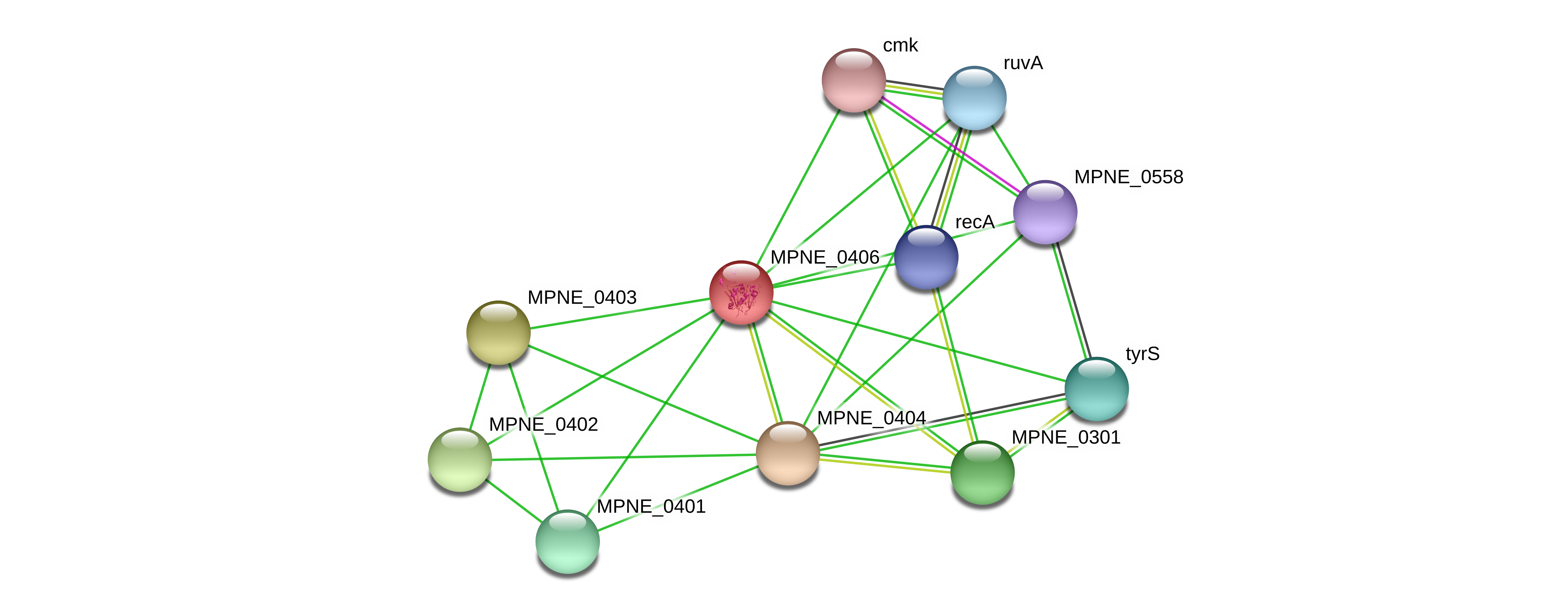

Supplement: Potential drug target dTIGR00282 family metallophosphoesterase and its protein interactions from STRING. [file rsos190907supp7.png]

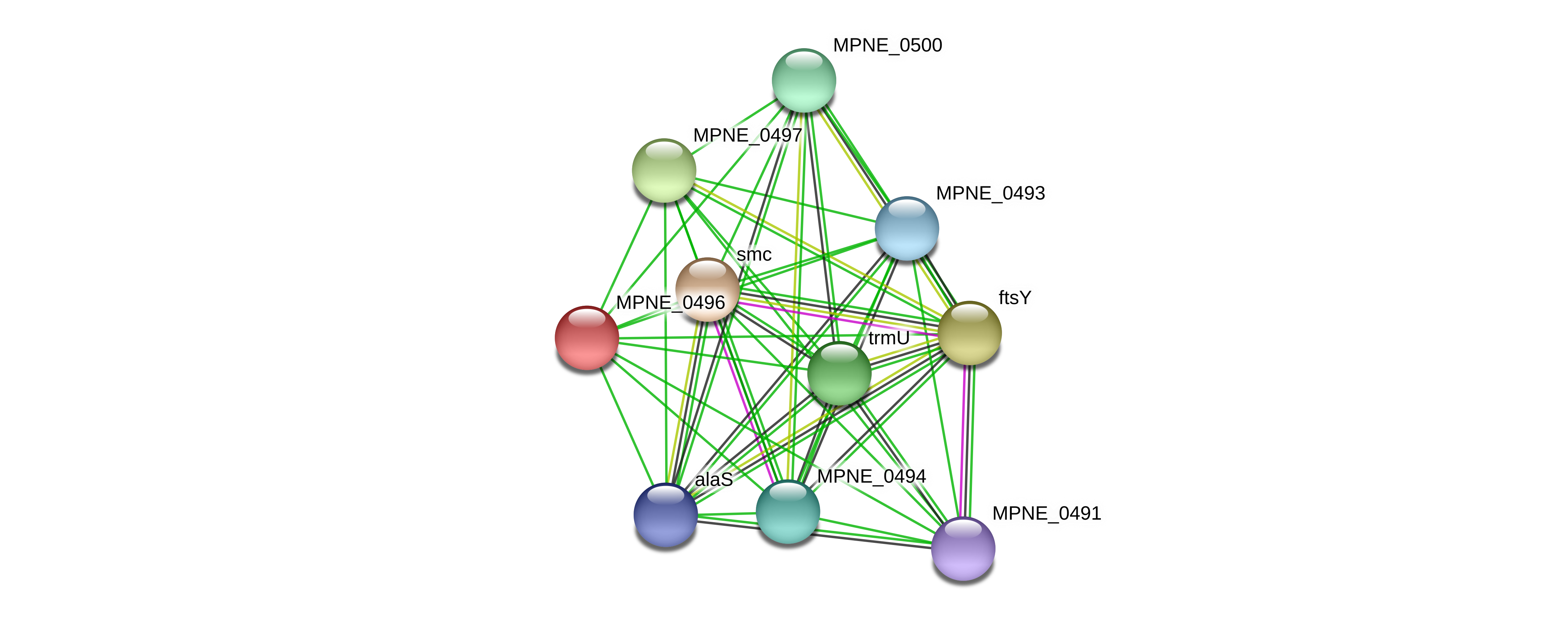

Supplement: Potential drug target Hypothetical protein MPN423and its protein interactions from STRING [file rsos190907supp9.png]

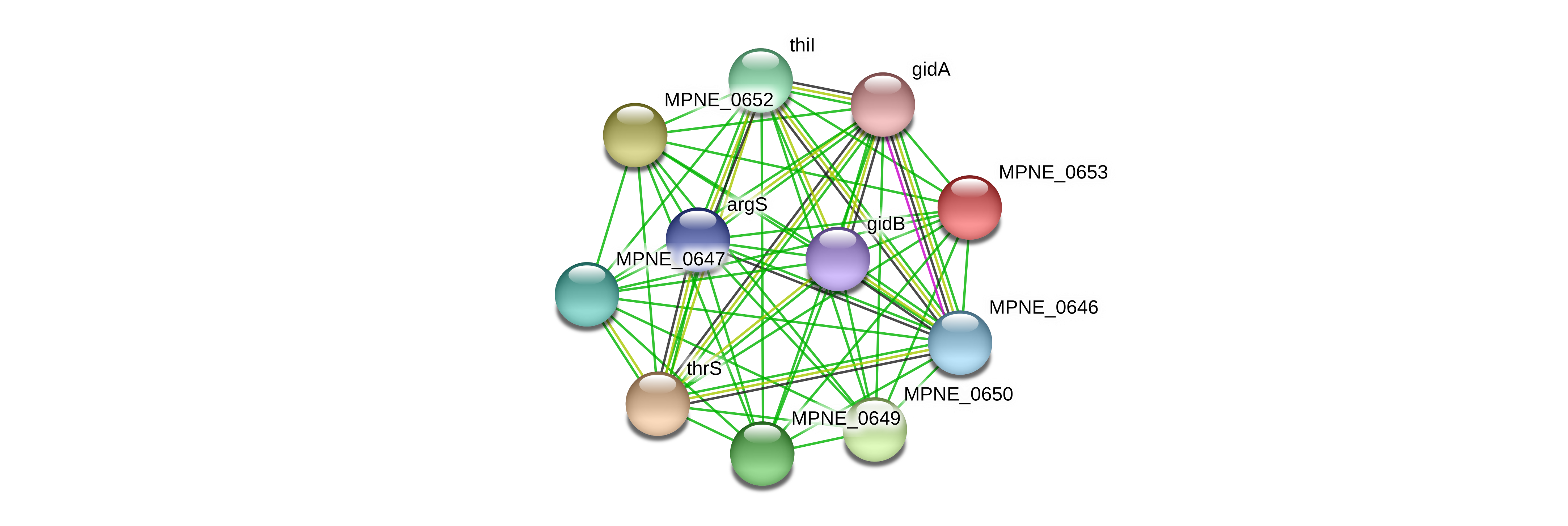

Supplement: Potential drug target Hypothetical protein and its protein interactions from STRING [file rsos190907supp11.png]

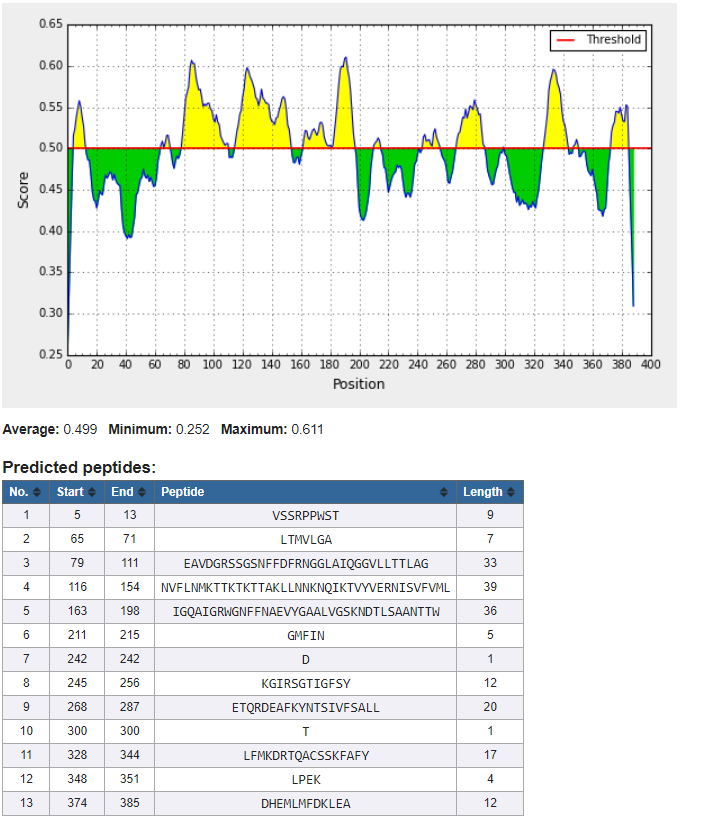

Supplement: Epitopes of the vaccine target WP_010874581.1 capable of being recognized by cell B [file rsos190907supp13.png]

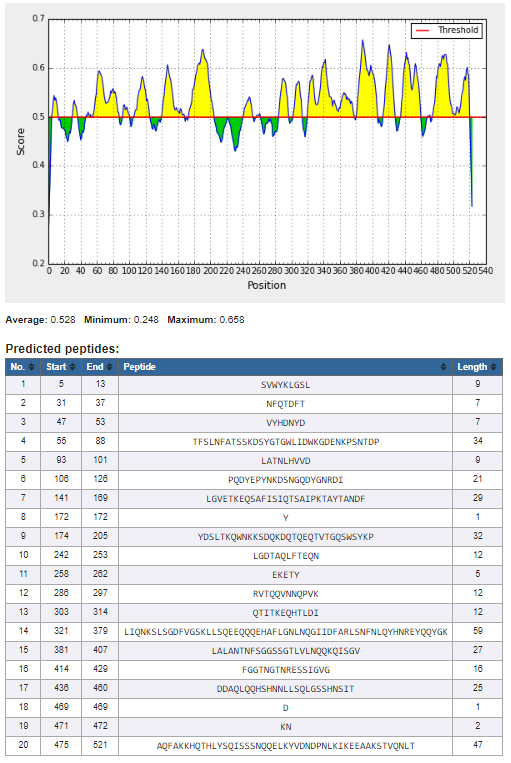

Supplement: Epitopes of the vaccine target WP_014574866.1 capable of being recognized by cell B [file rsos190907supp14.png]

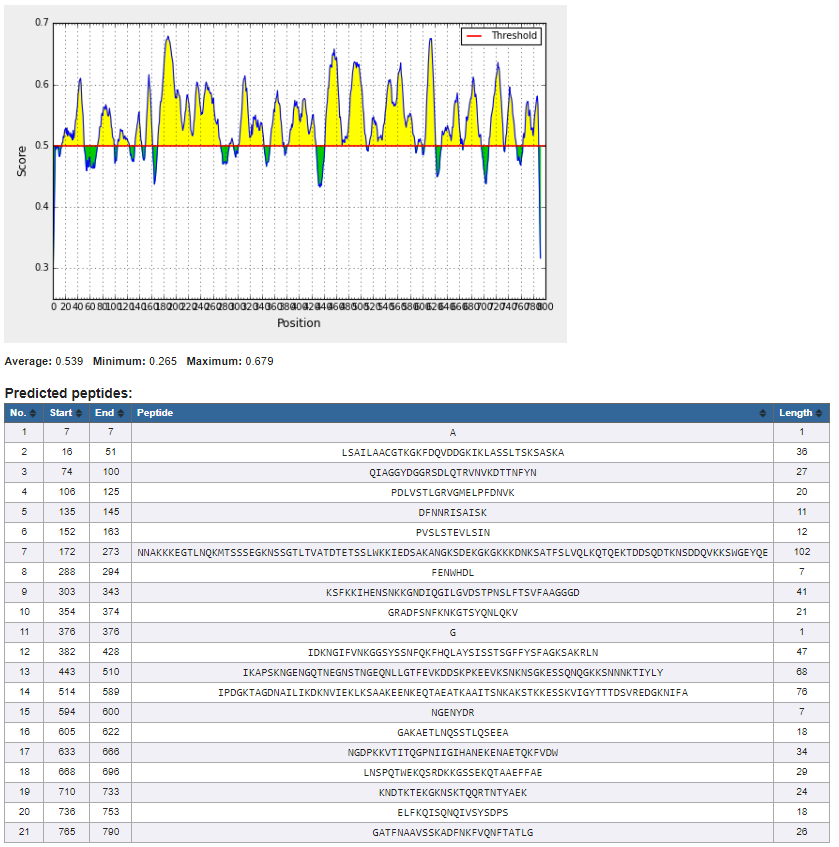

Supplement: Epitopes of the vaccine target WP_010874862.1 capable of being recognized by cell B [file rsos190907supp15.png]

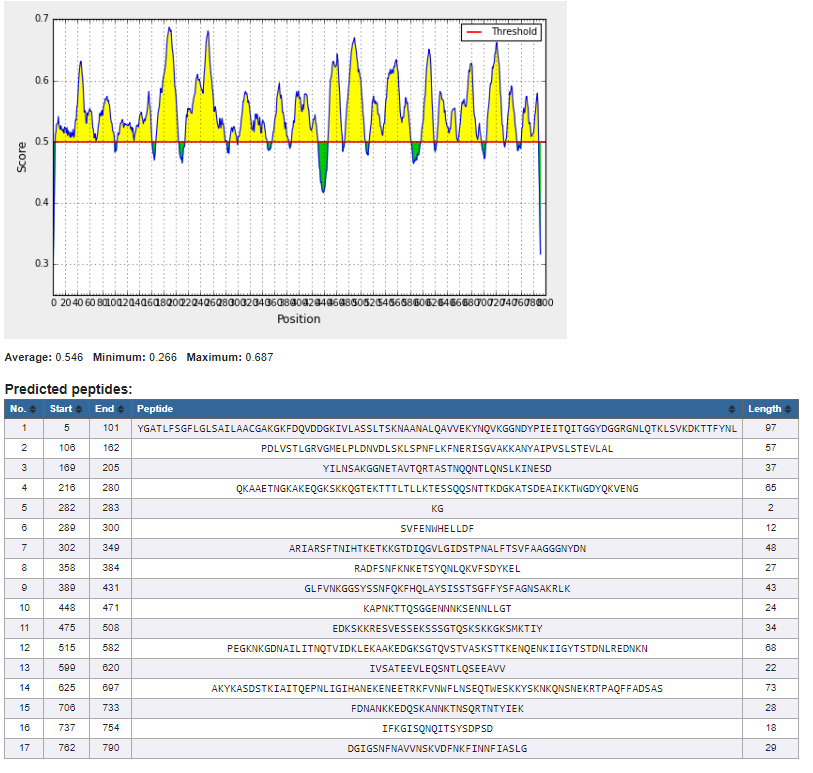

Supplement: Epitopes of the vaccine target WP_014325486.1 capable of being recognized by cell B. [file rsos190907supp16.png]

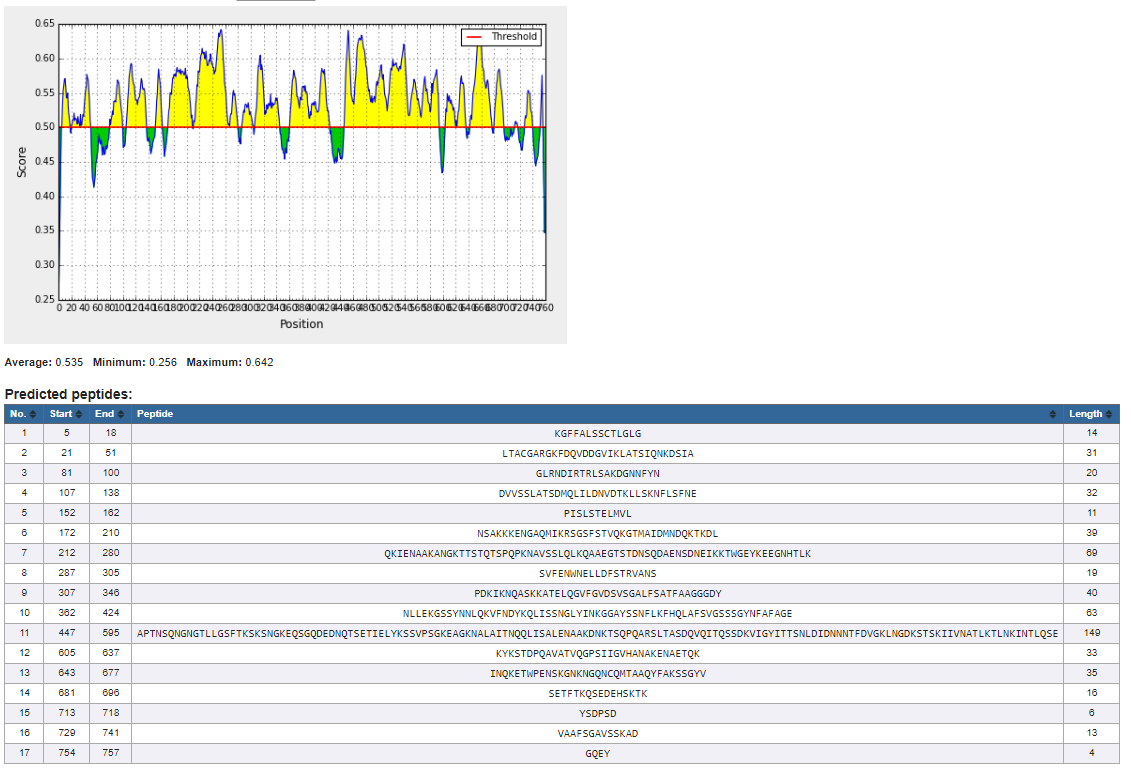

Supplement: Epitopes of the vaccine target WP_014325517.1 capable of being recognized by cell B. [file rsos190907supp17.png]

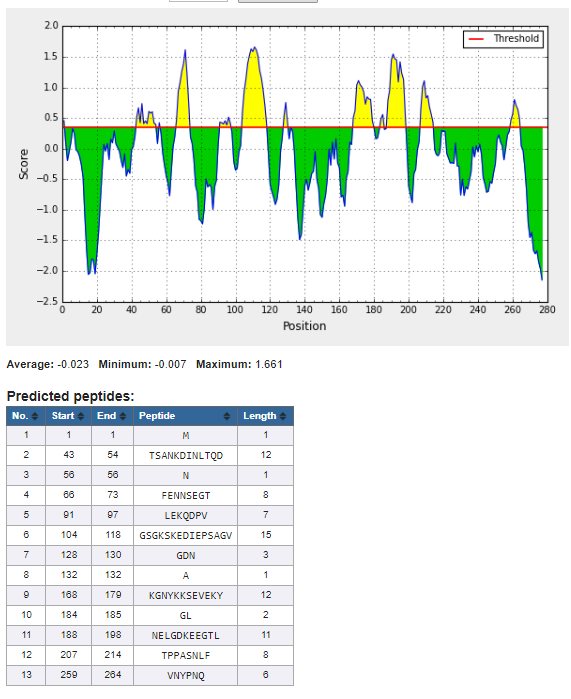

Supplement: Epitopes of the vaccine target WP_014325659.1 capable of being recognized by cell. B [file rsos190907supp18.png]

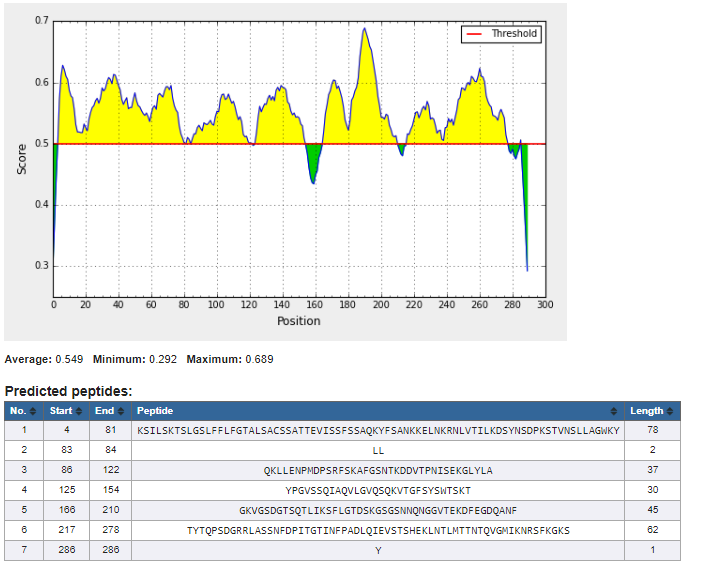

Supplement: Epitopes of the vaccine target WP_14325660.1 capable of being recognized by cell B. [file rsos190907supp19.png]

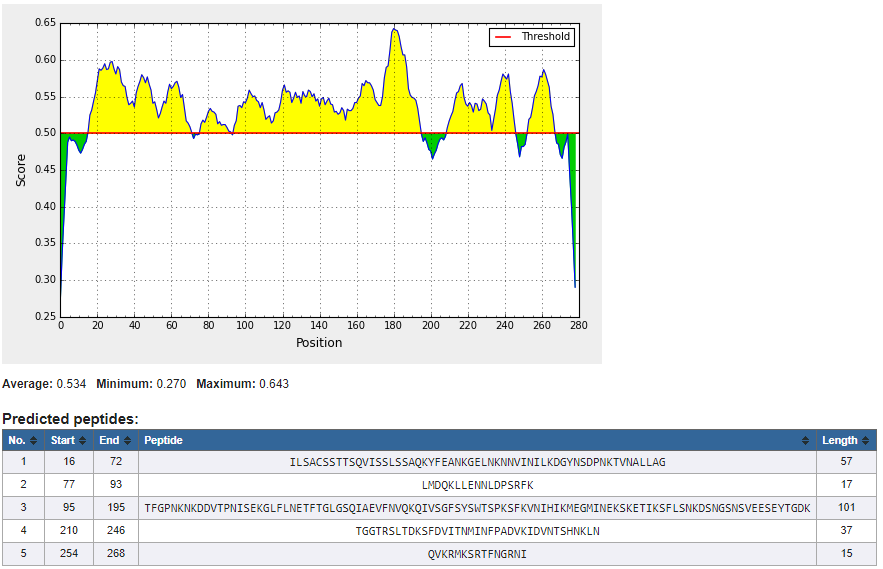

Supplement: Epitopes of the vaccine target WP_010874999.1 capable of being recognized by cell B [file rsos190907supp20.png]

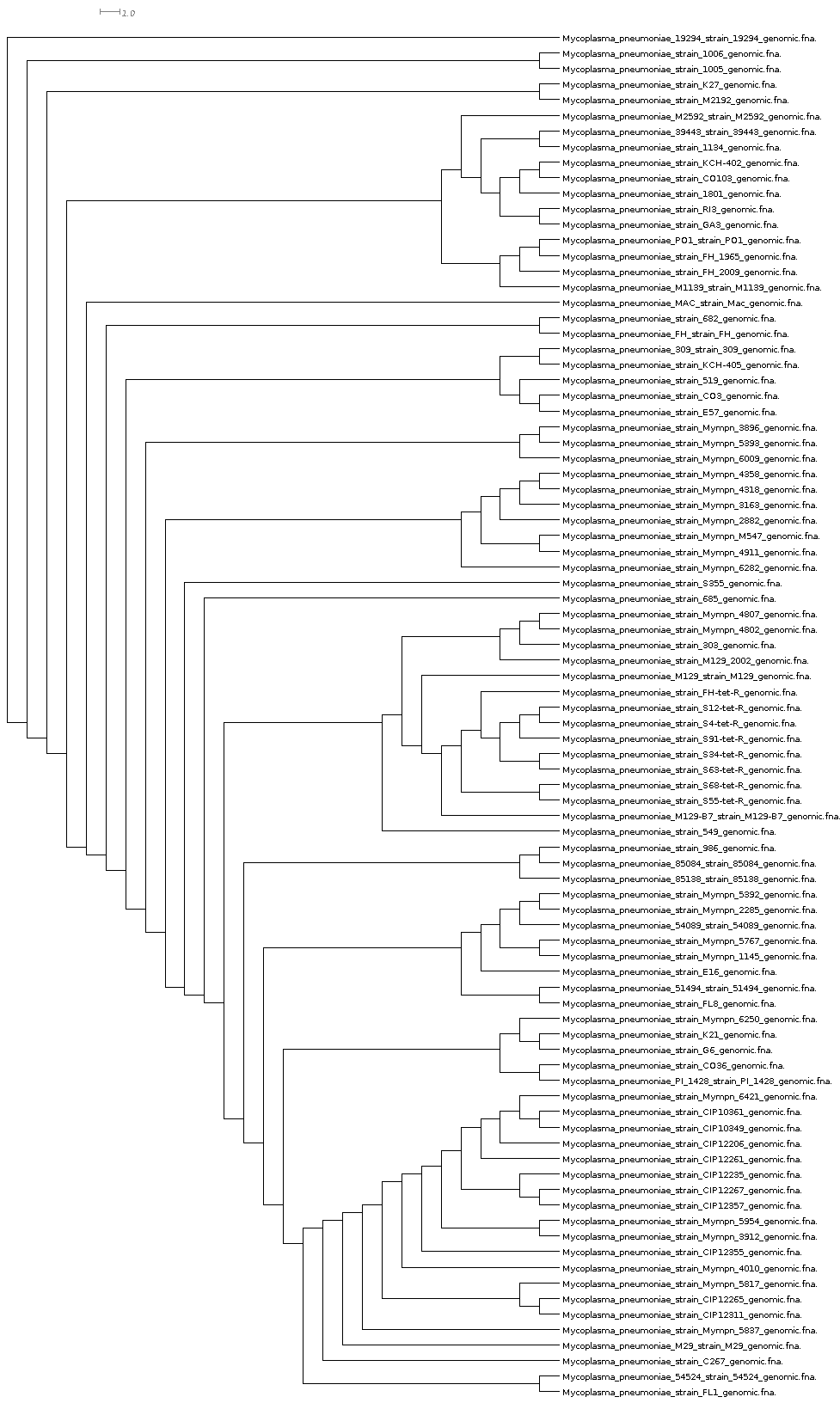

Supplement: Phylogenetic tree correlating the evolutive distance among the strains of M. pneumonia [file rsos190907supp22.png]
