# Supplementary material for: Reverse vaccinology and subtractive genomics reveal new therapeutic targets against Mycoplasma pneumoniae: a causative agent of pneumonia
Source: R Soc Open Sci. 2019 Jul 31;6(7):190907. doi: 10.1098/rsos.190907 (PMC6689572; doi:10.1098/rsos.190907)
Supplement: Top 10 Molecules from Virtual Screening for each Identified Targets [file rsos190907supp12.docx]

Top 10 Molecules from Virtual Screening for each Identified Targets.

| **ZINC Compound ID** |  | **Autodock vina Binding Affinity** | **No of H-bond/Residues** |
| --- | --- | --- | --- |
|  | **30S ribosome-binding factor (WP_010874513.1)** | | |
| ZINC04259381 |  | -10.5 | 3/ASN18, ARG15 |
| ZINC04259578 |  | -8.5 | 3/ALA78 |
| ZINC04259719 |  | -6.0 | 2/ARG75 |
| ZINC04235426 |  | -9.7 | 2/HIS37, LEU40 |
| ZINC05396219 |  | -10.5 | 1/ASN11 |
| ZINC20503175 |  | -7.2 | 1/ASN27 |
| ZINC04259499 |  | -9.1 | 1/THR32 |
| ZINC08300280 |  | -6.6 | 1/THR29 |
| ZINC31154666 |  | -8.5 | 2/ARG75 |
|  | **division/cell wall cluster transcriptional repressor MraZ (WP_010874670.1)** | | |
| ZINC04237100 |  | -9.4 | 1/GLN54 |
| ZINC08635277 |  | -8.5 | 1/ASP64 |
| ZINC04237105 |  | -10.0 | 1/ARG43 |
| ZINC04258871 |  | -10.4 | 2/ARG43, GLN54 |
| ZINC04235924 |  | -10.2 | 1/ARG34 |
| ZINC04237101 |  | -10.7 |  |
| ZINC04237091 |  | -9.0 | 1/ASN38 |
| ZINC04260408 |  | -9.5 | 1/ARG43 |
| 1ZINC04222214 |  | -10.6 | 1/CYS39 |
| ZINC04270981 |  | -10.7 | 2/ARG34, GLU41 |
|  | **dTIGR00282 family metallophosphoesterase (WP_010874705.1)** | | |
| ZINC05396856 |  | -8.5 | 1/LYS49 |
| ZINC04259703 |  | -8.9 | 3/LYS49, ASN71 |
| ZINC04235924 |  | -9.5 | 1/ASN71 |
| ZINC08300419 |  | -8.7 | 1/LYS139 |
| ZINC04237082 |  | -8.5 | 1/ASN71 |
| ZINC04259578 |  | -9.7 | 1/ASN71 |
| ZINC20503625 |  | -8.2 | 1/ASN71 |
| ZINC04270628 |  | -9.5 | 3/LYS196, GLY174, THR177 |
| ZINC04259588 |  | -9.6 | 1/ASN71 |
| ZINC04277685 |  | -9.6 | 1/ARG264 |
|  | **Hypothetical protein (WP_010874779.1 )** | | |
| ZINC08300419 |  | -11.9 | 1/CYS90 |
| ZINC05415832 |  | -11.1 | 1/PHE93 |
| ZINC05415069 |  | -12.3 | 1/LYS108 |
| ZINC04236421 |  | -10.5 | 1/SER51 |
| ZINC04235924 |  | -11.0 | 1/CYS89 |
| ZINC15709489 |  | -11.1 | 1/CYS89 |
| ZINC04237100 |  | -11.5 | 1/TYR115 |
| ZINC08635277 |  | -11.4 | 1/TYR115 |
| ZINC20503551 |  | -11.1 | 1/TYR115 |
| ZINC20503308 |  | -9.9 | 1/CYS89 |
|  | **Hypothetical protein (WP_014325598.1)** | | |
| ZINC04235924 |  | -11.8 | 1/TYR143 |
| ZINC04235884 |  | -11.6 | 1/SER43 |
| ZINC15672005 |  | -10.2 | 2/GLU49, LYS45 |
| ZINC04235972 |  | -11.5 | 1/GLU145 |
| ZINC04258871 |  | -9.5 | 1/HIS36 |
| ZINC04235928 |  | -10.6 | 1/SER43 |
| ZINC04236030 |  | -10.3 | 2/LYS45, TYR154 |
| ZINC04235880 |  | -10.1 | 1/ARG39 |
| ZINC04235930 |  | -10.8 | 2/SER43, ASN148 |
| ZINC03839937 |  | -9.4 | 1/LYS62 |
